# Concrete Crack Identification Using a UAV Incorporating Hybrid Image Processing

**DOI:** 10.3390/s17092052

**Published:** 2017-09-07

**Authors:** Hyunjun Kim, Junhwa Lee, Eunjong Ahn, Soojin Cho, Myoungsu Shin, Sung-Han Sim

**Affiliations:** 1 School of Urban and Environmental Engineering, Ulsan National Institute of Science and Technology (UNIST), Ulsan 44919, Korea; guswns3@unist.ac.kr (H.K.); lee.junhwa@unist.ac.kr (J.L.); eunjong@unist.ac.kr (E.A.); msshin@unist.ac.kr (M.S.); 2 Department of Civil Engineering, University of Seoul, Seoul 02504, Korea; soojin@uos.ac.kr

**Keywords:** concrete structure, crack identification, digital image processing, structural health monitoring, unmanned aerial vehicle

## Abstract

Crack assessment is an essential process in the maintenance of concrete structures. In general, concrete cracks are inspected by manual visual observation of the surface, which is intrinsically subjective as it depends on the experience of inspectors. Further, it is time-consuming, expensive, and often unsafe when inaccessible structural members are to be assessed. Unmanned aerial vehicle (UAV) technologies combined with digital image processing have recently been applied to crack assessment to overcome the drawbacks of manual visual inspection. However, identification of crack information in terms of width and length has not been fully explored in the UAV-based applications, because of the absence of distance measurement and tailored image processing. This paper presents a crack identification strategy that combines hybrid image processing with UAV technology. Equipped with a camera, an ultrasonic displacement sensor, and a WiFi module, the system provides the image of cracks and the associated working distance from a target structure on demand. The obtained information is subsequently processed by hybrid image binarization to estimate the crack width accurately while minimizing the loss of the crack length information. The proposed system has shown to successfully measure cracks thicker than 0.1 mm with the maximum length estimation error of 7.3%.

## 1. Introduction

Concrete is one of the most widely used materials for civil infrastructure such as bridges, buildings, and nuclear power plants because of its cost-effectiveness and convenience for molding into desired shapes. However, concrete structures inevitably suffer from cracks caused by creep, shrinkage, and loading that potentially degrade structural soundness. Indeed, cracks are an important indicator of structural health; consequently, crack monitoring is considered an essential maintenance process for civil infrastructure.

In general, cracks on concrete structures are inspected by manual visual observation of the surface. The observed information, including the length, direction, and width of the cracks are tracked over time to evaluate the current condition of the structure and anticipate crack growth, which is used to assist with maintenance plans. Although manual visual inspection is the most common practice applied to monitor concrete cracks, this method is intrinsically inefficient as it is time-consuming, expensive, and even unsafe for inspectors in the case of inaccessible structural members.

Recent advances in unmanned aerial vehicle (UAV) technologies have produced low-cost and high-mobility UAVs, rapidly broadening their real-world civil engineering application [1,2,3,4,5,6,7]. For example, aerial images taken by UAVs have been used to construct three-dimensional structural models [8,9,10,11], evaluate road conditions [12,13,14], and conduct traffic surveillance and management [15,16,17]. Furthermore, the use of UAVs in conjunction with digital image processing has also shown great potential to overcome the disadvantages of visual inspection for concrete crack monitoring [18,19,20,21,22]. UAVs enable the taking of images in proximity to surface cracks in full-scale civil engineering structures, facilitating better crack identification results. However, to the best of our knowledge, quantitative assessment of the crack width and length has not been reported in a UAV-based application.

Various image processing techniques have been implemented for effectively extracting the crack information from images. For example, edge detection has been utilized to provide a boundary between a crack and its background [23,24,25] and image binarization to transform the crack and background into black and white pixels [26,27]. In addition, mathematical morphology has been implemented to improve overall shape of cracks in images [28,29]. Cha et al. [30] used a deep learning approach to determine the existence of cracks from a concrete surface image; edge detection algorithms were further applied to localize cracks for width estimation. A wide variety of image processing algorithms for crack identifications are summarized in [31].

Image binarization is known to be one of the most commonly used image processing methods. In the binarization process, crack objects are identified by categorizing the pixels whose values are less than a specified threshold into black. Thus, the binary objects considered cracks can be used to determine the crack width and length with an associated working distance. However, although image binarization is useful for separating cracks and backgrounds, the crack assessment is difficult to standardize due to a high dependence of binarization on the parameters determined by users. Kim et al. [32] conducted parametric analysis to determine the optimal parameters of binarization for accurate crack width estimation. However, they found that using binarization with the optimal parameters often resulted in them being unable to find small cracks in blurred images. Thus, finding cracks with accurate width and complete length information is challenging in image processing. Indeed, existing UAV-based crack identification approaches have not conducted quantitative assessment of both crack width and length.

This paper presents a hybrid image processing-based crack identification using UAV. The UAV-based system designed in this study is capable of image and distance sensing, as well as wireless communication, which makes it possible to control sensing and data transmission while the UAV is in the air. The obtained information is subsequently processed by the proposed hybrid image processing to identify crack width accurately while minimizing the loss of crack length. The performance of the crack identification strategy using UAV is experimentally validated using a concrete wall with various types of cracks.

## 2. Background

### 2.1. Crack Width Estimation via Image Binarization

**Figure 1:**
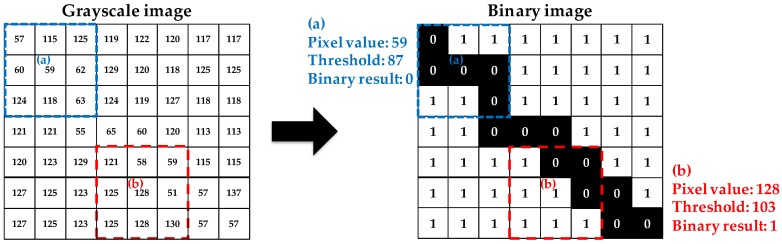
Demonstration of the binarization process in 3 × 3 windows (thresholds are determined simply using mean values) [32].

The first step in crack width calculation based on image binarization is to convert the RGB image to grayscale. Subsequently, a threshold is calculated using the statistical properties of the grayscale values (e.g., mean and standard deviation) to categorize each grayscale pixel into black and white. For example, as shown in Figure 1, the thresholds in each pixel are evaluated in 3 × 3 windows. When the grayscale pixel value is lower than the corresponding threshold, such as in pixel (a) in Figure 1, the binarization result is zero (black). In contrast, the binarization result for pixel (b) in Figure 1 is one (white) because the pixel value is higher than the threshold.

A wide variety of image binarization methods have been developed primarily for text detection from images [33,34,35,36,37]. Sauvola’s method [35], one of the most widely used binarization methods, is specifically designed to identify text from noisy backgrounds. Sauvola’s method is carefully selected in this work because concrete cracks have a similar shape to text and the crack images also have noisy backgrounds due to aggregates, dust, shadows, and holes. In Sauvola’s method, the threshold is calculated using Equation (1):

(1) $$T=m\left[1+k\left(\frac{s}{R}-1\right)\right]$$

where *m* is the mean in each selected window, *s* is the standard deviation, *R* is the dynamic range for normalizing *s*, and *k* is the sensitivity to control the contribution of the statistical parameters. Since the thresholds highly depend on two user-defined parameters of window size and sensitivity, these parameters should be appropriately selected for accurate binarization results.

**Figure 2:**
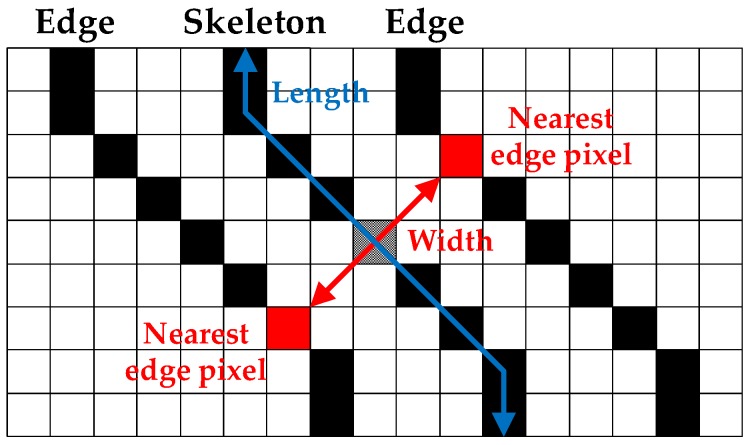
Illustration of crack width and length calculation.

The binary images are subsequently processed by skeleton and edge detection and crack width calculation. Each group of connected black pixels, representing a crack segment, is decomposed into a skeleton and edges using the thinning method [38] and edge detection method [39], respectively. The skeleton is a group of central pixels of a crack segment with crack length and direction information, and the edges are two collections of outer pixels of the crack segment containing the width information (see Figure 2). To obtain the crack width for a certain pixel in the skeleton, the crack direction is identified based on the connectivity of the pixel and its adjacent pixels; subsequently, edge pixels nearest to the skeleton pixel are sought in the skeleton direction. The directional information is particularly necessary when the skeleton is bent, as the nearest edge pixel could be located in a wrong direction. The crack width is obtained as the distance between both edge pixels nearest to the skeleton pixel. It is then converted to metric using the following pinhole camera equation [32]:

(2) $$W_{r}=D_{p}W_{p}=\frac{10D_{w}}{P_{c}L_{f}}W_{p}$$

where *W_r_* is the real crack width in metric (mm), *D_p_* is the resolution of the imaging system, *W_p_* is the obtained crack width in pixel, *D_w_* is the working distance in mm, *P_c_* is the pixels per centimeter of the used camera sensor, and *L_f_* is the focal length of the camera in mm.

### 2.2. Issues in Image Binarization for Crack Identification

**Figure 3:**
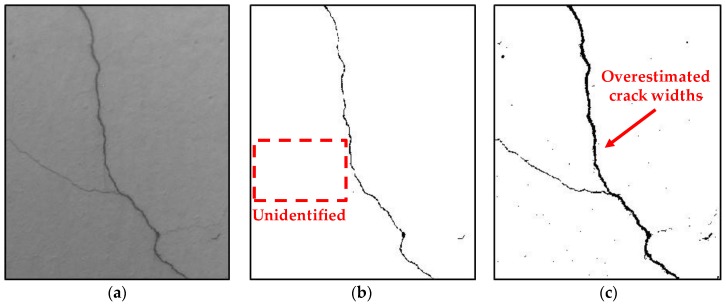
Binarization result of Sauvola’s method from different window sizes and sensitivities: (**a**) original grayscale image; (**b**) 80 × 80 window and sensitivity of 0.3 (Some cracks are unidentified); and (**c**) 100 × 100 window and sensitivity of 0.1 (the crack width is overestimated).

Image binarization has to be cautiously applied to crack assessment because of its dependence on user-defined parameters. Image binarization is capable of effectively extracting crack information using a simple equation, such as Equation (1); however, the measurement accuracy of the detected crack width and length is generally affected by the image binarization parameters initially provided by the user. For example, as shown in Figure 3, when the same crack image is processed using the image binarization method, the obtained crack information can be different according to the used binarization parameters. In Figure 3b, while the crack width is accurately detected, some small cracks are unidentified. Whereas the existence of cracks is better detected as shown in Figure 3c, the crack width is overestimated. Thus, it is difficult to exactly obtain crack widths while minimizing the loss of length information using a specific set of binarization parameters. Note that the non-crack elements marked as black pixels, as shown in Figure 3b,c, can be removed based on their eccentricity and size.

## 3. Hardware Configuration for Crack Information Acquisition

**Figure 4:**
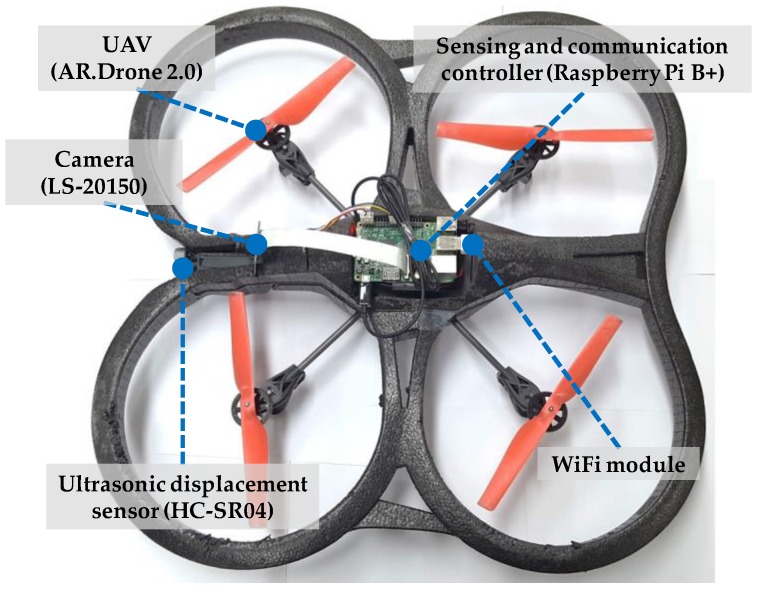
UAV-based system for crack information acquisition.

The UAV-based prototype used in this study is designed to effectively acquire necessary data for crack identification [40]. The prototype is developed based on an off-the-shelf quadcopter, the Parrot AR.Drone 2.0 (Parrot, Paris, France), because of its cost-effectiveness, high mobility, and convenient control interface using a smartphone. The UAV is equipped with four essential components: a sensing and communication controller, a camera, an ultrasonic displacement sensor, and a WiFi module, as shown in Figure 4 and Table 1.

Raspberry Pi B+, a low-cost low-power single-board computer running Linux, is utilized to control sensing and communication. The Raspberry Pi is interfaced with the camera, the displacement sensor, and the USB WiFi module. The Raspberry Pi B+ takes images using the camera, measures the working distance between the camera and the concrete structure, and is controlled by and sends data to a remote computer using the WiFi module. The USB WiFi module mounted on the Raspberry Pi provides wireless connection between the UAV-based system and the operator’s computer through a WiFi router. Remote access to the Raspberry Pi of the UAV-based system allows operators to acquire image and distance information when desired and to wirelessly transmit the acquired data. The operator can monitor the video being taken by the camera, and instantly acquire image and distance data that are wirelessly transmitted to the operator’s computer.

The camera module (LS-20150) and the ultrasonic displacement sensor (HC-SR04) provide crack images and the corresponding working distances, which are required to determine crack sizes. In previous studies [18,19,20,21,22], quantitative assessment of cracks was ineffective or unavailable because measured distance information was not obtained. The camera module has a maximum resolution of five million square pixels, which is adequate for crack image acquisition, despite its light weight of 10.3 g. The small focal ratio (F-number) of the camera module enables the highest shutter speed; thus, any effect of the movement and vibration of the UAV on the crack images is minimized. The obtained crack images can be blurred because of the intrinsic vibration and movement of the UAV and, thus, the image blur is an important issue that has to be addressed. The image blur is closely related to the exposure time of the camera shutter when capturing images, and can be alleviated by increasing the shutter speed, resulting in low brightness. Thus, an optimal shutter speed has to be selected considering the trade-off; a shutter speed of 1/1000 s for the LS-20150 camera module is sufficiently fast to produce bright and clear images in most cases.

All the components of the proposed system are selected to be low-cost and lightweight. The total weight of the sensing and communication components (i.e., Raspberry Pi with the camera, the ultrasonic displacement sensor, and the WiFi module) is approximately 60 g, which does not significantly affect the flight of the UAV. To further reduce the weight, the sensing and communication components are designed to share the UAV’s battery. The power consumption of the Raspberry Pi is approximately 2 W, which is significantly less than that of the UAV (70 W).

## 4. Hybrid Image Processing Strategy for Crack Identification

In addition to the hardware described in Section III, an image processing strategy tailored to the UAV-based system is developed in this study. The main idea underlying the strategy is to consider a combination of different sets of binarization parameters for accurately extracting the crack width while minimizing loss of length. The proposed hybrid image processing strategy comprises two stages: (1) image pre-processing to prepare the image for further analysis and (2) crack width estimation using the hybrid approach.

### 4.1. Image Pre-Processing

**Figure 5:**
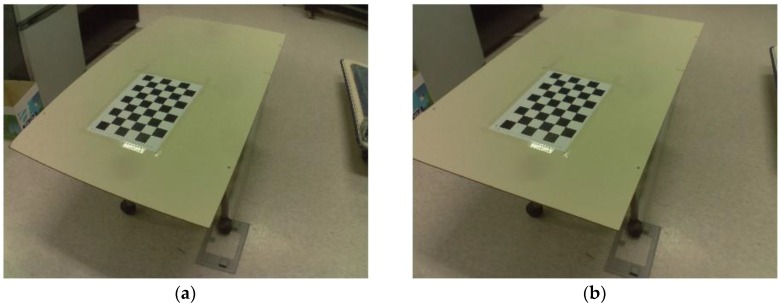
Illustrative example for the image calibration algorithm: (**a**) image distortion resulted from wide-angle lens; and (**b**) image undistortion using image calibration.

The image pre-processing stage consists of two steps: (1) image undistortion and (2) conversion from color image to grayscale image. As shown in Figure 5a, the selected low-cost lens produces a distorted image, from which crack width estimation can be seriously impaired. To calibrate this image [41], a black and white checker board is captured using the camera with different angles and distances, to estimate the intrinsic and extrinsic parameters. After determining the camera parameters, the image taken by that camera is undistorted, as shown in Figure 5b. Subsequently, the calibrated image is converted to grayscale, as the image colors are unnecessary for identifying cracks.

### 4.2. Crack Width Estimation

Hybrid image processing is applied to the pre-processed image to determine crack width and length accurately. As stated in Section 2, a set of binarization parameters of sensitivity and window size is difficult to estimate crack width and length simultaneously. Thus, the hybrid approach employs two sets of binarization parameters, each of which provides the least error in width and length estimations, respectively. Let *P_w_* and *P_l_* designate these two sets:

- *P_w_*: optimal parameters minimizing estimation errors in crack width; and
- *P_l_*: optimal parameters minimizing estimation errors in crack length.

*P_w_* and *P_l_* are then separately employed to generate two binary images using Sauvola’s method. *P_l_* inevitably results in a higher threshold than that of *P_w_* to convert more pixels to crack elements.

The binary images are subsequently processed using the steps for skeleton and edge detection and crack width calculation, as described in Section 2. Following width estimation, the obtained width information is recorded in each location of the skeleton pixels. The sets of skeleton pixels and their related crack width are defined as follows:

- *S_w_*: set of skeleton pixels obtained using *P_w_*;
- *S_l_*: set of skeleton pixels obtained using *P_l_*; and
- *w*(*P*, *S*): crack width at location S obtained using *P*.

where *P* is either *P_w_* or *P_l_*, and *S* is a set of skeleton pixels. Selecting *P_w_* to produce the accurate crack width of *w*(*P_w_*, *S_w_*) results in more unidentified crack elements than *P_l_*. Thus, *S_w_* is a subset of *S_l_*, because the obtained thresholds of *P_l_* are greater than those of *P_w_*. However, the calculated widths obtained using *P_l_* (i.e., *w*(*P_l_*, *S_l_*)) are overestimated owing to the high thresholds.

**Figure 6:**
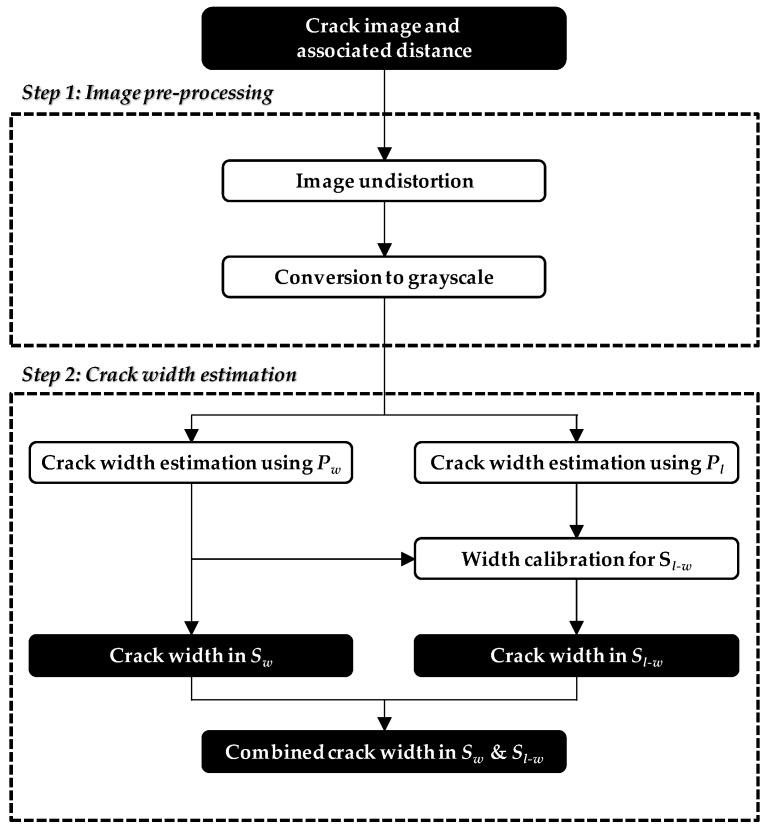
Schematic outline of the hybrid image processing strategy.

The final crack widths are a combination of *w*(*P_w_*, *S_w_*) and *w*(*P_l_*, *S_l_ − S_w_*), which are the crack widths using *P_w_* at *S_w_* and *P_l_* at *S_l_ − S_w_*, respectively. The overestimated crack width *w*(*P_l_*, *S_l_ − S_w_*) has to be corrected, which enables the simultaneous generation of accurate crack width and length. The calibration for the overestimated *w*(*P_l_*, *S_l_ − S_w_*) can be performed by utilizing the ratio of *w*(*P_w_*, *S_w_*) and *w*(*P_l_*, *S_w_*), as defined in Equation (3):

(3) $$\alpha =\frac{1}{N}\overset{N}{\underset{i=1}{\sum }}\frac{w_{i}\left(P_{w},S_{w}\right)}{w_{i}\left(P_{l},S_{w}\right)}$$

where *α* is the calibration factor, *N* is the number of skeleton pixels in *S_w_*, and *w_i_* is the crack width at the *i*th skeleton pixel. The calibration factor *α* is then multiplied by *w*(*P_l_*, *S_l_ − S_w_*) to correct the overestimation. The crack widths of *w*(*P_w_*, *S_w_*) and *αw*(*P_l_*, *S_l_ − S_w_*) are combined to provide complete width and length information in *S_l_*. The overall procedure of the proposed hybrid image processing strategy is summarized in Figure 6.

## 5. Experimental Validation

A field testing is conducted to demonstrate the validity of the proposed hybrid image processing in conjunction with the UAV. First, parametric analysis was conducted to determine the two sets of optimal parameters, *P_w_* and *P_l_*. Subsequently, crack identification by hybrid image processing was performed using crack images obtained from the UAV-based system.

### 5.1. Determination of Optimal Parameters

**Figure 7:**
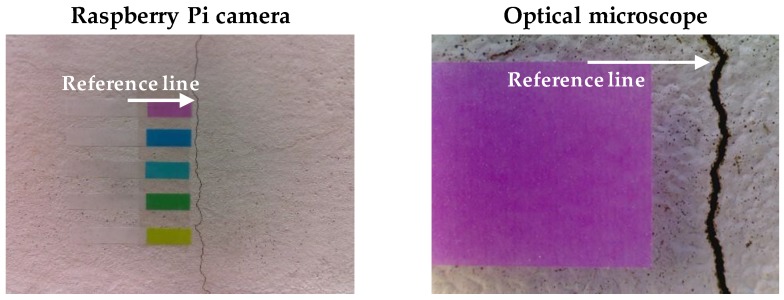
Comparison of detected crack widths to references.

In the parametric analysis, 20 crack images with different surface textures, crack widths, lengths, directions, and sizes, were prepared to address various concrete conditions. The collected image pool was processed using Sauvola’s method with a wide range of binarization parameters. Then, crack width and length information was obtained using the procedure presented in Section 2. Figure 7 shows a typical image included in the image pool. An optical microscope was used to measure reference widths at the specific locations, where the color targets were attached, as shown in Figure 7.

Two cost functions were defined to determine the optimal parameters in terms of crack width and length. The first cost function J*_w_*, for optimal width, is defined as:

(4) $$J_{w}=\frac{1}{N_{p}N_{t}}\overset{N_{p}}{\underset{i=1}{\sum }}\sqrt{\overset{N_{t}}{\underset{j=1}{\sum }}{\left(\frac{w_{e}-w_{m}}{w_{m}}\right)}^{2}}$$

where *N_p_* is the total number of images in the pool, *N_t_* is the number of color targets, *w_e_* is the estimated crack width from Sauvola’s method, and *w_m_* is the width measured by the optical microscope. The second cost function J*_l_*, for optimal length, is defined as:

(5) $$J_{l}=\frac{1}{N_{p}}\overset{N_{p}}{\underset{i=1}{\sum }}\left(\left|\frac{l_{e}-l_{t}}{l_{t}}\right|+\frac{C_{d}}{C_{t}}\right)$$

where *l_e_* is the estimated crack length from Sauvola’s method, and *l_t_* is the total length verified visually in the grayscale image. *C_d_* and *C_t_* are the numbers of detected crack and total pixels in the entire binary image, respectively. The second term in Equation (5) prevents all the pixels from being converted into cracks.

**Figure 8:**
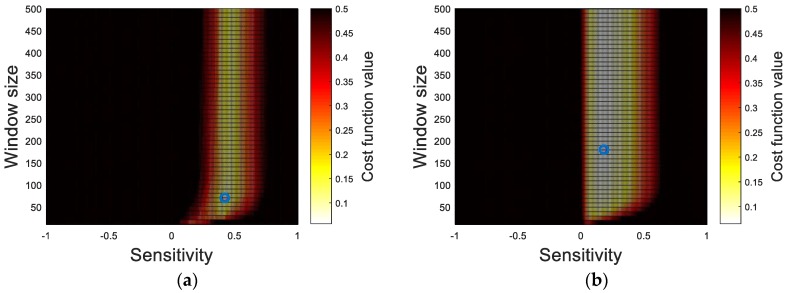
Cost functions in terms of window size and sensitivity: (**a**) J*_w_*; and (**b**) J*_l_*.

The cost function values of each crack information set (i.e., crack width and length) are analyzed with respect to the binarization parameters, as shown in Figure 8, to determine the two sets of optimal parameters. From the results, the sensitivity is observed as a governing factor rather than the window size in both cost functions. The lowest cost function values in each case, marked as the blue circles, are selected to determine the optimal parameters summarized in Table 2.

### 5.2. Crack Identification Using the Hybrid Image Processing Strategy

**Figure 9:**
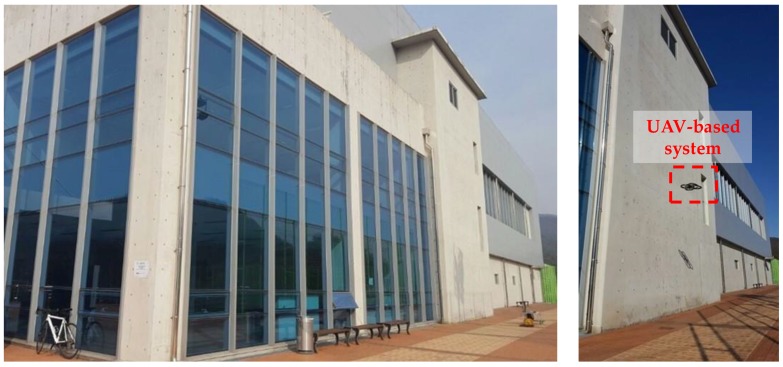
Experimental validation using concrete wall.

Field testing was conducted on a concrete wall in the gymnasium building on the UNIST campus (see Figure 9). The concrete wall has cracks with diverse shapes and sizes due to creep, shrinkage, and loads. The UAV-based system acquired the crack images and the corresponding working distances using the Raspberry Pi camera and the displacement sensor, while flying in front of the concrete wall. Note that the crack widths were also measured using the optical microscope as the reference to compare with those from the UAV-based crack identification system.

**Figure 10:**
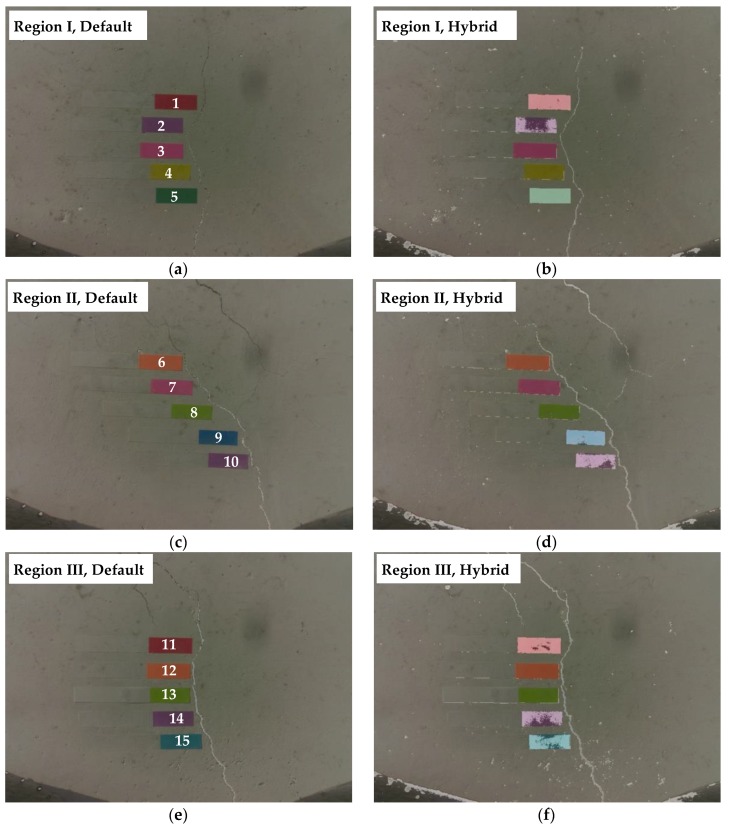
Crack identification results: (**a**) region I, Sauvola’s method with default parameter; (**b**) region I, hybrid method; (**c**) region II, Sauvola’s method with default parameter; (**d**) region II, hybrid method; (**e**) region III, Sauvola’s method with default parameter; and (**f**) region III, hybrid method.

The crack identification using the hybrid image processing is applied to the captured images. To validate the performance of the proposed hybrid method, the binarization results were compared with the results of Sauvola’s method with default parameters adopted from [35]. As shown in Figure 10, it is clear that the hybrid method better located the cracks than with only the default parameters. Note that the black objects on the bottom side of the captured images are the part of the UAV. Quantitative comparisons of crack widths and lengths are conducted at a total of 15 points in three crack regions as presented in Table 3 and Table 4. With the default values, cracks with widths less than 0.25 mm were typically unidentified or underestimated. In contrast, the hybrid method measured all range of crack widths reliably, because small cracks unidentified by Sauvola’s method using *P_w_* can also be detected and calibrated accurately.

Cracks thinner than about 0.1 mm, which can be seen with the naked eye, are not found even with the hybrid image processing as shown in Figure 10. Although accuracy of the image processing is not as good as the manual visual observation, its efficiency in terms of identification time would be critical particularly when a number of crack images are to be processed. The accuracy-related issue can be resolved by preparing appropriate hardware of camera and lens that can detect cracks thicker than the minimum width of interest for maintenance purposes.

### 5.3. Discussion

The minimum detectable crack size is reported as the most important parameter of the inspection method [42]. From the experimental validation on a concrete wall, the proposed approach has shown to accurately measure cracks thicker than 0.1 mm with the maximum length estimation error of 7.3%. According to American Concrete Institute ACI 224R-90 [43], tolerable crack width is designed with regard to the exposure condition as shown in Table 5. Therefore, the proposed approach can be applied except for water retaining structures.

When a UAV is used with computer vision, parallax during image acquisition is an important issue [44]. As this study primarily focuses on developing the image processing approach for identification of crack width and length, crack images are taken while UAV is flying near the concrete surface to minimize the effect of parallax.

## 6. Conclusions

This paper presented the crack identification using the hybrid image processing strategy with a UAV. A prototype of the UAV-based system was built using a Raspberry Pi connected to a camera, an ultrasonic displacement sensor, and a WiFi module. The Raspberry Pi controlled sensing and wireless communication, providing crack images with associated distances on demand. The obtained information was subsequently processed by the hybrid image processing method using two sets of optimal parameters *P_w_* and *P_l_*, to accurately detect crack widths while minimizing loss of crack lengths. The results of the experimental evaluation can be summarized as follows:

(1) While the crack widths less than 0.25 mm were typically unidentified or underestimated in case of the default values, the proposed hybrid method measured all ranges of crack widths reliably.
(2) The maximum length estimation errors were 7.3% and 52.3% for the hybrid method and Sauvola’s binarization with the default parameters, respectively, proving significant performance improvement by the hybrid method.

Consequently, the results of experimental evaluation on a concrete wall show that the proposed UAV and hybrid image processing-based crack identification strategy effectively and reliably identifies cracks.

## Figures and Tables

**Table 1:** System components.

Component	Model	Specification
UAV	Parrot AR.Drone 2.0	Dimensions: 58 cm × 13 cm × 58 cm
		Weight: 1.8 kg
Sensing and communication controller	Raspberry Pi B+	CPU: 700 MHz single-core
		Memory: 512 MB
		Weight: 45 g
Camera	LS-20150	Resolution: 2592 pixels × 1944 pixels
		Focal length: 2.8 mm
		F-number: 2.8
		Weight: 10.3 g
Ultrasonic displacement sensor	HC-SR04	Measurable distance: 2 cm–4 m
		Resolution 0.3 cm
		Weight: 8.5 g

**Table 2:** Optimal crack width and length parameters.

	Sensitivity	Window Size	Cost Function
*P_w_*	0.42	70	0.057
*P_l_*	0.18	180	0.065

**Table 3:** Comparison of obtained crack lengths.

Region	Total Crack Length Calculation (mm)		
	Default (Error)	Hybrid (Error)	Manual
I	37.49 (52.3%)	72.86 (7.3%)	78.57
II	79.18 (42.0%)	128.75 (5.7%)	136.50
III	95.01 (18.8%)	115.99 (0.9%)	117.02

**Table 4:** Comparison of obtained crack widths.

Region	Location	Crack Width Calculation (mm)		
		Default (Difference)	Hybrid (Difference)	Microscope
I	1	N/A *	0.14 (0.02)	0.12
	2	N/A *	0.14 (0.02)	0.12
	3	0.15 (−0.07)	0.20 (−0.02)	0.22
	4	0.15 (−0.08)	0.20 (−0.03)	0.23
	5	N/A *	0.13 (−0.01)	0.14
II	6	N/A *	0.22 (0.03)	0.19
	7	0.20 (−0.03)	0.25 (0.02)	0.23
	8	0.30 (−0.02)	0.30 (−0.02)	0.32
	9	0.25 (0.01)	0.25 (0.01)	0.24
	10	0.35 (−0.04)	0.40 (0.01)	0.39
III	11	N/A *	0.22 (0.03)	0.19
	12	0.49 (−0.04)	0.49 (−0.04)	0.53
	13	0.49 (−0.01)	0.49 (−0.01)	0.50
	14	0.59 (0.04)	0.59 (0.04)	0.55
	15	0.59 (0.04)	0.59 (0.04)	0.55

* The crack is unidentified.

**Table 5:** Tolerable crack widths for exposure conditions of ACI 224R-90 [43].

Exposure Condition	Tolerable Crack Width (mm)
Dry air protective membrane	<0.40
Humidity, moist air, soil	<0.30
Deicing chemicals	<0.18
Seawater and seawater spray; Wetting and drying	<0.15
Water retaining structures	<0.10

## References

[1] Colomina I., Molina P. (2014). Unmanned aerial systems for photogrammetry and remote sensing: A review. ISPRS J. Photogramm. Remote Sens..

[2] Nex F., Remondino F. (2014). UAV for 3D mapping applications: A review. Appl. Geomat..

[3] Campos I.S., Nascimento E.R., Freitas G.M., Chaimowicz L. (2016). A height estimation approach for terrain following flights from monocular vision. Sensors.

[4] Chen S., Laefer D.F., Mangina E. (2016). State of technology review of civilian UAVs. Recent Pat. Eng..

[5] Gonzalez L.F., Montes G.A., Puig E., Johnson S., Mengersen K., Gaston K.J. (2016). Unmanned Aerial Vehicles (UAVs) and artificial intelligence revolutionizing wildlife monitoring and conservation. Sensors.

[6] Sampedro C., Bavle H., Sanchez-Lopez J.L., Fernández R.A.S., Rodríguez-Ramos A., Molina M., Campoy P. A flexible and dynamic mission planning architecture for UAV swarm coordination. Proceedings of the International Conference on Unmanned Aircraft Systems (ICUAS).

[7] Vetrella A.R., Fasano G., Accardo D., Moccia A. (2016). Differential GNSS and vision-based tracking to improve navigation performance in cooperative multi-UAV systems. Sensors.

[8] Zongjian L.I.N. (2008). UAV for mapping—Low altitude photogrammetric survey. Int. Arch. Photogramm. Remote Sens. Spat. Inf. Sci..

[9] Remondino F., Barazzetti L., Nex F., Scaioni M., Sarazzi D. (2011). UAV photogrammetry for mapping and 3d modeling–current status and future perspectives. Int. Arch. Photogramm. Remote Sens. Spat. Inf. Sci..

[10] Siebert S., Teizer J. (2014). Mobile 3D mapping for surveying earthwork projects using an Unmanned Aerial Vehicle (UAV) system. Autom. Constr..

[11] Byrne J., O’Keeffe E., Lennon D., Laefer D.F. (2017). 3D Reconstructions using unstabilized video footage from an unmanned aerial vehicle. J. Imaging.

[12] Chen S.E., Rice C., Boyle C., Hauser E. (2011). Small-format aerial photography for highway-bridge monitoring. J. Perform. Constr. Facil..

[13] Zhang C., Elaksher A. (2012). An unmanned aerial vehicle-based imaging system for 3D measurement of unpaved road surface distresses1. Comput.-Aided Civ. Infrastruct. Eng..

[14] Díaz-Vilariño L., González-Jorge H., Martínez-Sánchez J., Bueno M., Arias P. (2016). Determining the limits of unmanned aerial photogrammetry for the evaluation of road runoff. Measurement.

[15] Srinivasan S., Latchman H., Shea J., Wong T., McNair J. Airborne traffic surveillance systems: Video surveillance of highway traffic. Proceedings of the ACM 2nd International Workshop on Video Surveillance and Sensor Networks.

[16] Puri A. (2005). A Survey of Unmanned Aerial Vehicles (UAV) for Traffic Surveillance.

[17] Heintz F., Rudol P., Doherty P. From images to traffic behavior-a uav tracking and monitoring application. Proceedings of the 10th International Conference on Information Fusion.

[18] Eschmann C., Kuo C.-M., Kuo C.-H., Boller C. (2013). High-resolution multisensor infrastructure inspection with unmanned aircraft systems. Int. Arch. Photogramm. Remote Sens. Spat. Inf. Sci..

[19] Choi S.-S., Kim E.-K. Building crack inspection using small UAV. Proceedings of the 17th International Conference on Advanced Communication Technology.

[20] Pereira F.C., Pereira C.E. (2015). Embedded image processing systems for automatic recognition of cracks using UAVs. IFAC-PapersOnLine.

[21] Sankarasrinivasan S., Balasubramanian E., Karthik K., Chandrasekar U., Gupta R. (2015). Health monitoring of civil structures with integrated UAV and image processing system. Procedia Comput. Sci..

[22] Ellenberg A., Kontsos A., Moon F., Bartoli I. (2016). Bridge related damage quantification using unmanned aerial vehicle imagery. Struct. Control Health Monit..

[23] Abdel-Qader I., Abudayyeh O., Kelly M.E. (2003). Analysis of edge-detection techniques for crack identification in bridges. J. Comput. Civ. Eng..

[24] Hutchinson T.C., Chen Z. (2006). Improved image analysis for evaluating concrete damage. J. Comput. Civ. Eng..

[25] Zhao H., Qin G., Wang X. Improvement of canny algorithm based on pavement edge detection. Proceedings of the 3rd International Congress on Image and Signal Processing.

[26] Liu Y., Cho S., Spencer B.F., Fan J. (2014). Automated assessment of cracks on concrete surfaces using adaptive digital image processing. Smart Struct. Syst..

[27] Liu Y.F., Cho S., Spencer B.F., Fan J. (2016). Concrete crack assessment using digital image processing and 3D scene reconstruction. J. Comput. Civ. Eng..

[28] Sinha S.K., Fieguth P.W. (2006). Segmentation of buried concrete pipe images. Autom. Constr..

[29] Giakoumis I., Nikolaidis N., Pitas I. (2006). Digital image processing techniques for the detection and removal of cracks in digitized paintings. IEEE Trans. Image Process..

[30] Cha Y.J., Choi W., Büyüköztürk O. (2017). Deep learning-based crack damage detection using convolutional neural networks. Comput.-Aided Civ. Infrastruct. Eng..

[31] Jahanshahi M.R., Kelly J.S., Masri S.F., Sukhatme G.S. (2009). A survey and evaluation of promising approaches for automatic image-based defect detection of bridge structures. Struct. Infrastruct. Eng..

[32] Kim H., Ahn E., Cho S., Shin M., Sim S.-H. (2017). Comparative analysis of image binarization methods for crack identification in concrete structures. Cem. Concr. Res..

[33] Bernsen J. Dynamic thresholding of grey-level images. Proceedings of the 8th International Conference on Pattern Recognition.

[34] Niblack W. (1986). An Introduction to Digital Image Processing.

[35] Sauvola J., Pietikäinen M. (2000). Adaptive document image binarization. Pattern Recognit..

[36] Wolf C., Jolion J.M. (2004). Extraction and recognition of artificial text in multimedia documents. Pattern Anal. Appl..

[37] Khurshid K., Siddiqi I., Faure C., Vincent N. Comparison of Niblack inspired binarization methods for ancient documents. Proceedings of the 16th International Conference on Document Recognition and Retrieval.

[38] Lam L., Lee S.-W., Suen C.Y. (1992). Thinning methodologies—A comprehensive survey. IEEE Trans. Pattern Anal. Mach. Intell..

[39] Canny J. (1986). A computational approach to edge detection. IEEE Trans. Pattern Anal. Mach. Intell..

[40] Kim H., Sim S.-H. Concrete crack assessment using unmanned aerial vehicle. Proceedings of the 24th Australasian Conference on the Mechanics of Structures and Materials.

[41] Bouguet J.Y. Camera Calibration Toolbox for Matlab. http://www.vision.caltech.edu/bouguetj/calib_doc/.

[42] Laefer D.F., Gannon J., Deely E. (2010). Reliability of crack detection methods for baseline condition assessments. J. Infrastruct. Syst..

[43] ACI 224R-90 (1990). Control of Cracking in Concrete Structures.

[44] Byrne J., Laefer D.F., O’Keeffe E. (2017). Maximizing feature detection in aerial unmanned aerial vehicle datasets. J. Appl. Remote Sens..

